# Estimating the incidence of tuberculosis cases reported at a tertiary hospital in Ghana: a time series model approach

**DOI:** 10.1186/s12889-018-6221-z

**Published:** 2018-11-26

**Authors:** George Aryee, Ernest Kwarteng, Raymond Essuman, Adwoa Nkansa Agyei, Samuel Kudzawu, Robert Djagbletey, Ebenezer Owusu Darkwa, Audrey Forson

**Affiliations:** 1 Department of Anaesthesia, School of Medicine and Dentistry, University of Ghana, Legon, Ghana; 2 Department of Medicine and Therapeutics, School of Medicine and Dentistry, University of Ghana, Legon, Ghana; 3 Department of Chest and Infectious Diseases, Korle-Bu Teaching Hospital, Accra, Ghana

**Keywords:** Tuberculosis, Time series, Incidence, Estimate, Forecast

## Abstract

**Background:**

The incidence of Tuberculosis (TB) differs among countries and contributes to morbidity and mortality especially in the developing countries. Trends and seasonal changes in the number of patients presenting with TB have been studied worldwide including sub-Saharan Africa. However, these changes are unknown at the Korle-Bu Teaching Hospital (KBTH). The aim of this study was to obtain a time series model to estimate the incidence of TB cases at the chest clinic of the Korle-Bu Teaching hospital.

**Methods:**

A time series analysis using a Box-Jenkins approach propounded as an autoregressive moving average (ARIMA) was conducted on the monthly TB cases reported at the KBTH from 2008 to 2017. Various models were stated and compared and the best was found to be based on the Akaike Information Criterion and Bayesian Information Criterion.

**Results:**

There was no evidence of obvious increasing or decreasing trend in the TB data. The log-transformed of the data achieved stationarity with fairly stable variations around the mean of the series. ARIMA (1, 0, 1) or ARMA (1,1) was obtained as the best model. The monthly forecasted values of the best model ranged from 53 to 55 for the year 2018; however, the best model does not always produce the best results with respect to the mean absolute and mean square errors.

**Conclusions:**

Irregular fluctuations were observed in the 10 -year data studied. The model equation to estimate the expected monthly TB cases at KBTH produced an AR coefficient of 0.971 plus an MA coefficient of − 0.826 with a constant value of 4.127. The result is important for developing a hypothesis to explain the dynamics of TB occurrence so as to outline prevention programmes, optimal use of resources and effective service delivery.

**Electronic supplementary material:**

The online version of this article (10.1186/s12889-018-6221-z) contains supplementary material, which is available to authorized users.

## Background

Tuberculosis (TB) is one of the infectious diseases distressing many countries widely and transmitted by the bacterium known as *Mycobacterium Tuberculosis* [1]. According to the World Health Organisation (WHO), “persons with TB bacteria have a 5-15% lifetime risk of falling ill with TB [2]; however, persons with compromised immune systems such as people living with HIV(PLWH), malnutrition or diabetes, and those with tobacco use have much higher risk of falling ill” [2]. The incidence of Tuberculosis varies among different countries worldwide. It is estimated that one-third(1/3) of the world’s population has been plague-ridden with the *M. tuberculosis*, particularly in the developing countries, as a major cause of morbidity and mortality worldwide [3, 4]. An estimated 9 million new cases of tuberculosis arise annually with an estimated 1.7million deaths globally [5]. In 2015, the highest number of new TB cases occurred in Asia (61%) whilst 26% in Africa and usually infect adults in their fecund years [2]. In Ghana, it is estimated that each year over 46,000 new cases of TB occur [6].

Globally, the annual TB incidence has decreased by an average of 1.5% since 2000 which needs to increase to a 4–5% yearly drop to attain the 2020 milestones of the End TB strategy. Between 2000 and 2015, an estimated 49 million lives were rescued as a result of TB diagnosis and treatment [2]. While efforts being made in dealing with the condition leading to a decline through various TB programmes and interventions, trends and seasonal models associated with the occurrence of TB have also been studied extensively [7–12]. In the Ashanti Region of Ghana, Gyasi-Agyei and colleagues [12] found that tuberculosis incidence studied can best be modelled with an autoregressive moving average [ARMA (1, 0) or AR (1)], and was predicted to be steady between April 2013 and April 2015. However, at the Korle-Bu Teaching Hospital, in the Greater Accra Region, there is a paucity of information regarding the trends and peaks period of reported TB cases referred to the facility. Trends in the incidence of TB has the propensity to impact significantly on planning and more efficient use of the facility’s resources as well as public health intervention programmes. Therefore, the aim of this study was to obtain a time series model to estimate the incidence of TB cases at the chest clinic of the Korle-Bu Teaching Hospital.

## Methods

### Study design and site

A time series analysis of time-dependent data comprising of 120 reported monthly TB cases from 2008 to 2017 at the chest unit of the Department of Medicine and Therapeutics, Korle-Bu Teaching Hospital (KBTH) was conducted. Korle-Bu Teaching Hospital is the largest and the premier teaching hospital in Ghana with a bed capacity of 2000 as at 2013. It is a major referral centre for the whole of Ghana and the West African Sub-region. The chest unit caters for patients with chest diseases such as Tuberculosis. The average number of patients with TB seen per month is seventy (70). Prior permission to use the data was obtained from the Chest clinic of the Korle-Bu Teaching Hospital. The study did not require ethical review because the used data never had identifiers nor anonymous human biological materials associated with them (The letter explaining this has been attached to the study).

### Data analysis

Data was inputted into Microsoft Excel 2013 and analysed in R statistical software version 3.3.2. Box-Jenkins time series approach put forward as Autoregressive Integrated Moving Average (ARIMA) model was employed for modelling. The Box-Jenkins methodology comprised model Identification, Parameter Estimation, Model Diagnostics and Forecasting [13]. Time series of the data was plotted for the period 2008–2017 to identify the various time series components in the data. The data were log-transformed and re-plotted. Stationarity was assessed and confirmed using the Augmented Dickey-Fuller (ADF) test on the transformed data. The series was judged stationary with the *p*-value of the ADF test ≤5% level of significance. An Autocorrelation Function (ACF) and Partial Autocorrelation Function (PACF) were plotted to obtain the orders *p* and *q* of AR and MA respectively. Upon determining the order of AR and MA terms, the model was obtained. The autoregressive model equation of order (*p*) is expressed as: *Y*_*t*_ = Φ_1_*Y*_*t* − 1_+_2_Φ*Y*_*t* − 2_ + . . ……… + Φ_*p*_*Y*_*t* − *p*_ + *w*_*t*_, where *Y*_*t*_ represents the current value of the series, *Y*_*t-1*,_………*Y*_*t-p*_ denotes the prior values of the same series whilst *w*_*t*_ is the white noise andΦ_1,…….,_ Φ_*p*_are the regression coefficients of the model.

The moving average model equation of order (*q*) is also written as:

*Y*_*t*_ = *w*_*t*_ + *ϕ*_1_*w*_*t* − 1_ + *ϕ*_2_*w*_*t* − 2_ + ……… + *ϕ*_*q*_*w*_*t* − *q*_, where *Y*_t_ denotes the current value of the series, *w*_t... ... *...*_ *w*_*t-q*_ are the white noise and *ϕ*_1_… *ϕ*_*q*_are the regression coefficients of the model.

Thus, the ARIMA model is given as *ϕ* (*B*) (*1* ‐ *B*)^*d*^*Y*_*t*_ = *θ* (*B*) *ω*_*t*_ where *ϕ* (*B*) is the operator for the AR term given as *ϕ* (*B*) = *1* ‐ *ϕ*_*1*_*B* − *ϕ*_2_*B*^2^ − … − *ϕ*_*P*_*B*^*P*^ and *θ* (*B*) is the operator for the MA term and is given as *θ* (*B*) = *1* + *θ*_*1*_*B* + *θ*_2_*B*^2^ + … + *θ*_*q*_*B*^*q*^. Where *p* and *q* represent the respective number of lags for the AR and MA terms and *d* represents the order of the integration term.

Whilst the ARMA model is a blend of both the AR with order *p* and MA with order *q* expressed in the equation: *Y*_*t*_ = Φ_1_*Y*_*t* − 1_ + Φ_2_*Y*_*t* − 2_ + . . ……… + Φ_*p*_*Y*_*t* − *p*_ + *w*_*t*_ + *ϕ*_1_*w*_*t* − 1_ + *ϕ*_2_*w*_*t* − 2_ + ……… + *ϕ*_*q*_*w*_*t* − *q*_.

The model obtained was compared to other ARIMA models. The model with the least Akaike Information Criterion (AIC) and Bayesian Information Criterion (BIC) was selected as the best model. Diagnostic tests were done on the best-chosen model by performing a residual analysis to determine the adequacy of the model; this was done by assessing the normality and independence of the residuals. The normality of the residuals was determined using the Quantile–Quantile (Q-Q) plot and confirmed by the Shapiro-Wilk’s test. Residual points found within the significant bounds of the ACF of the residual plot determined the independence of the residuals and confirmed by the Ljung-Box test. The best model was used to forecast the estimated number of monthly TB cases. A *p*-value ≥5% level of significance of the Shapiro-Wilk’s and Ljung-Box tests was considered statistically significant. Forecasting errors such as the Mean Square Error (MSE) and Mean Absolute Error (MAE) of the specified models were determined to ascertain the accuracy of the model for prediction a year ahead. The model with the minimum errors was considered accurate for prediction but the best model does may not necessarily give the best forecasting errors.

## Results

**Fig. 1:**
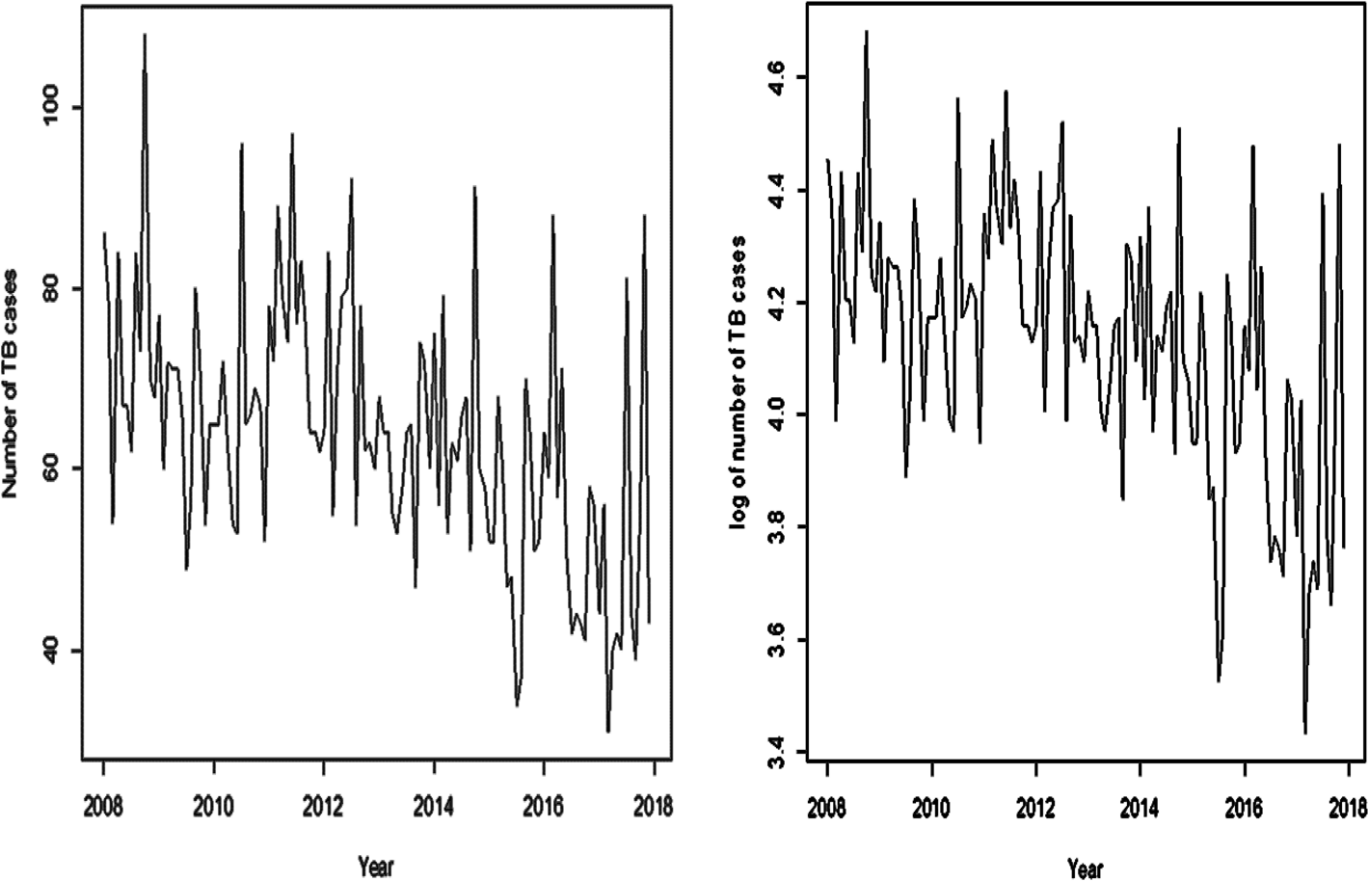
Time plot of the series actual data (left Graph) and log-transformed of the actual data (right Graph) over the period 2008–2016

The monthly TB time-dependent data consisted of 120 data points for the period (2008–2017) with a total number of 7676 cases. The time plot of the data showed fairly the same level from 2008 to 2012 but began to decrease slowly with several irregular fluctuations within the series (left plot in Fig. 1) with a peak in October and trough in March. However, the log-transformed of the time series data achieved quite a number of stable fluctuations as shown in Fig. 1(right plot).

From Fig. 1(right plot), it was found that the log-transformed of the series achieved stationarity. The Augmented Dickey Fuller test statistically confirmed stationarity of the series (*ADF = − 3.84, p-value = 0.020*).

**Fig. 2:**
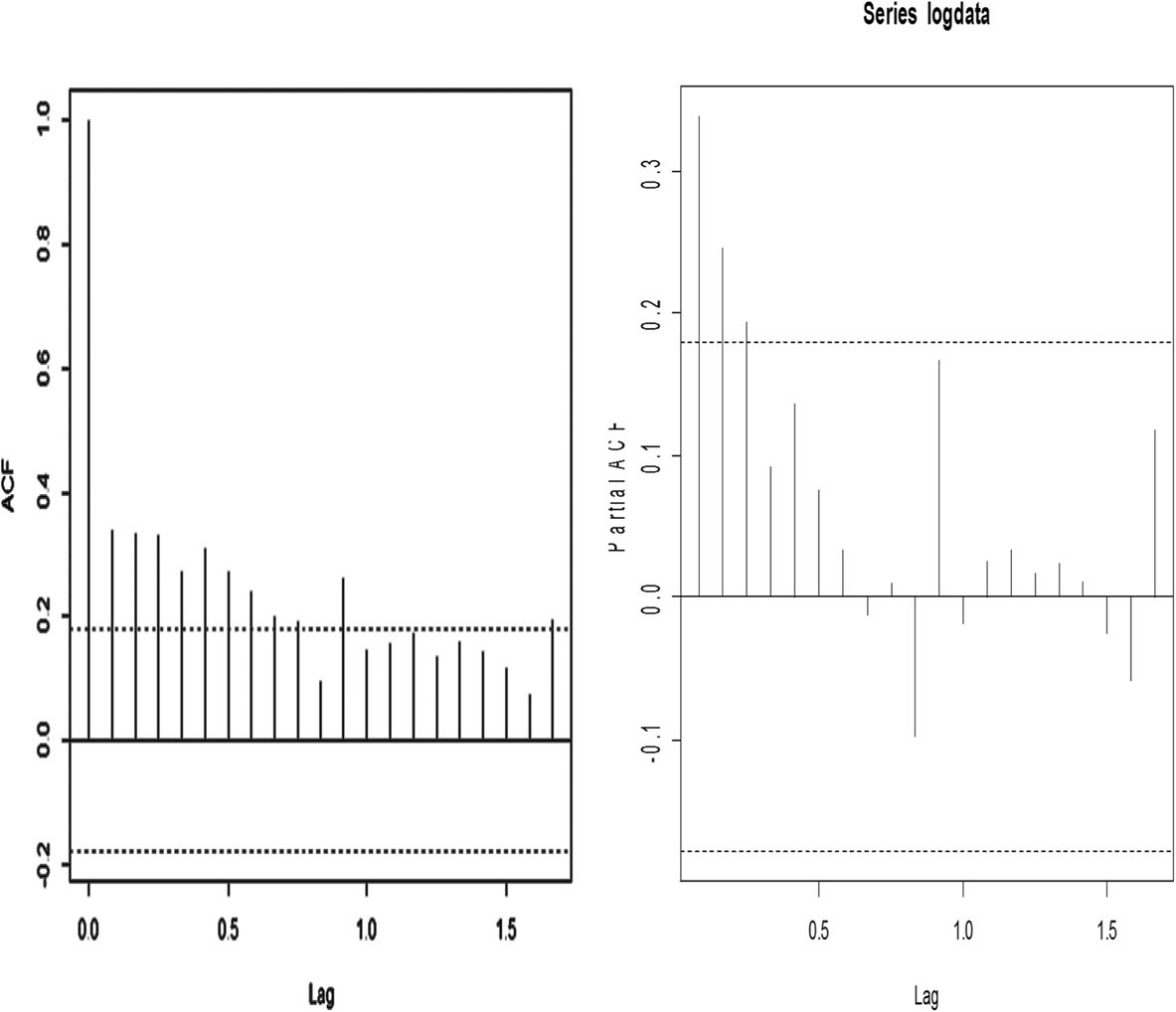
Correlogram plot of the ACF (left Graph) and PACF (right Graph) for the log-transformed of the actual data at various lags. The horizontal dash lines in the ACF and PACF are the significant bounds

The ACF indicated spikes at different lags (i.e 0,1, 2, 3, 4 etc.) above the significant bounds and the PACF also indicated spikes at lags 1 and 2 above the significant bounds (Fig. 2).

From the plots of the ACF and PACF (Fig. 2), the model ARIMA (1, 0, 1) was selected with 0.953 and − 0.784 as the regression coefficients of AR (1) and MA (1) respectively. The estimated intercept of the model was 4.137.

Other models formulated were compared to the empirical model ARIMA (1, 0, 1) as shown in Table 1 using their AICs and BICs.

**Table 1:** Comparison between formulated models and Ideal Model

No.	Models	AIC	BIC
1.	ARIMA(1,0,1)	− 32.76	−26.61
2.	ARIMA(1,0,2)	− 30.81	− 16.87
3.	ARIMA(2,0,1)	−30.81	−16.88
4.	ARIMA(3,0,1)	− 28.90	−12.17
5.	ARIMA(3,0,2)	−27.68	−8.17
6.	ARIMA(0,0,1)	−13.02	−4.66
7.	ARIMA(1,0,0)	−17.97	−9.6
8.	ARIMA(0,0,2)	−16.71	−5.56
9.	SARIMA (1,0,1)*(1,0,1)_12_	−30.55	−13.83
10.	SARIMA (1,0,2)*(0,0,1)_12_	− 29.19	−12.46
11.	SARIMA (2,0,1)*(1,0,1)_12_	−28.67	−9.16
12.	SARIMA (2,0,1)*(1,0,0)_12_	−29.07	−12.35

From Table 1, the best model was selected based on the minimum AIC and BIC values. It was found that ARIMA (1, 0, 1) had the minimum AIC and BIC. Hence ARIMA (1, 0, 1), the empirical model was selected as the best model among the other models formulated.

### ARIMA (1, 0, 1) with zero mean model diagnostics

**Fig. 3:**
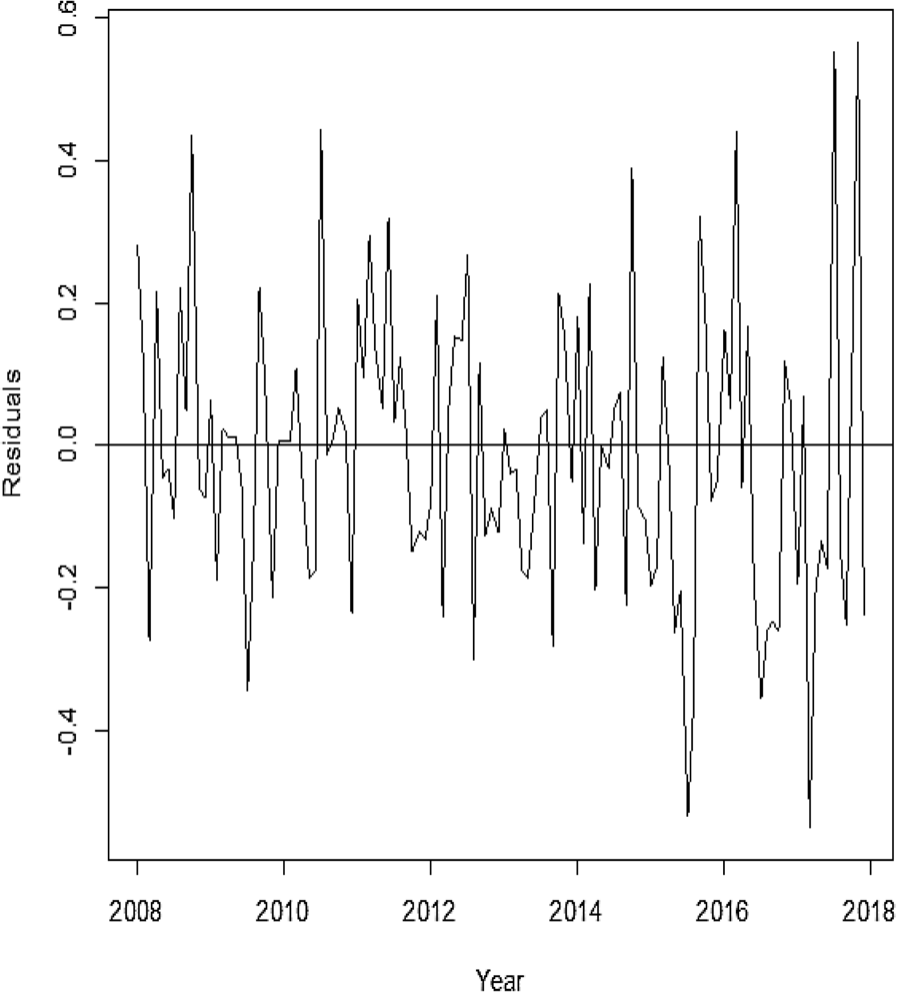
Plot of the standard residuals of the actual data period (2008–2017) and the forecasted figures for 2018 of the obtained model [ARIMA (1,0,1)] around a horizontal constant line

Figure 3 showed that a plot of the model residuals was fairly constant.

**Fig. 4:**
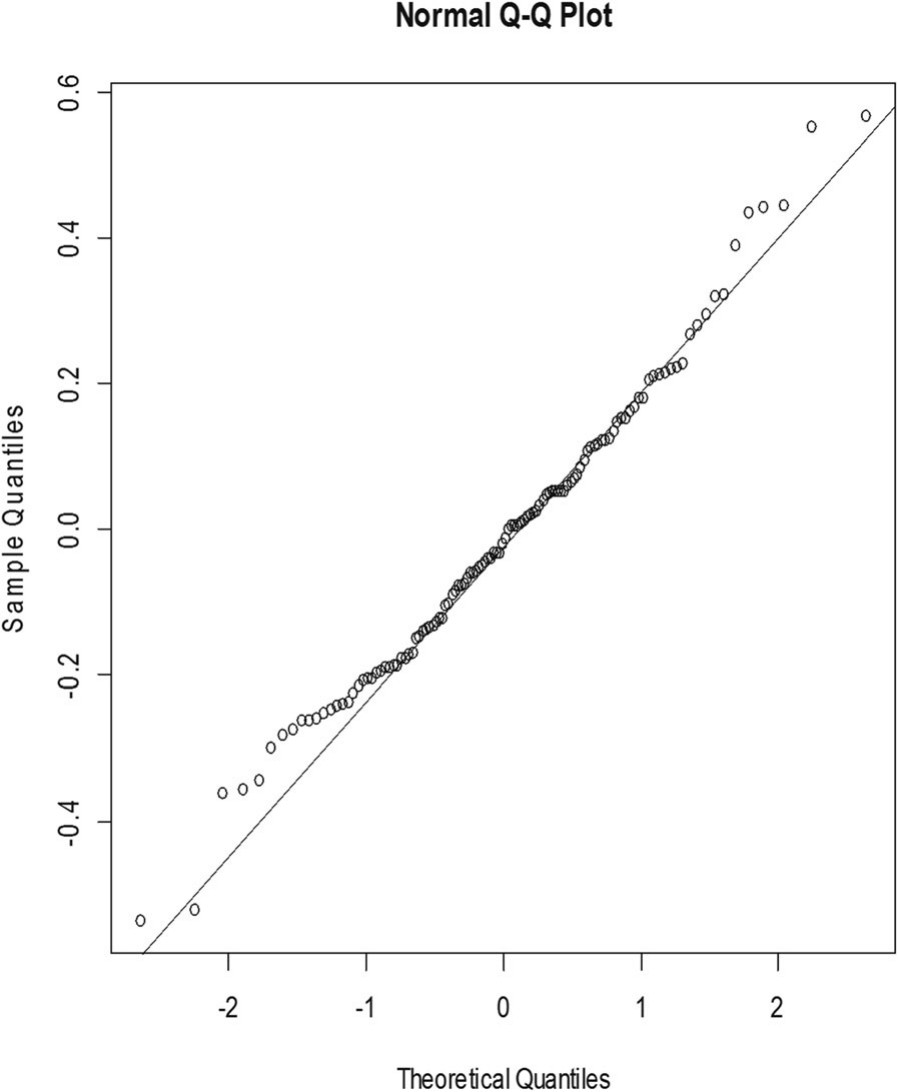
Quantile-Quantile plot of the model residuals. The data points around the diagonal line (line of symmetry) in the plot represent the model residuals to assess if the model residuals are from a normal distribution

The Q-Q plot in Fig. 4 also showed the model residuals were normally distributed as most of the residual points were closed to the normal line. The Shapiro-Wilk’s normality test confirmed normality of the residuals *(W = 0.986, p-value = 0.270).*

**Fig. 5:**
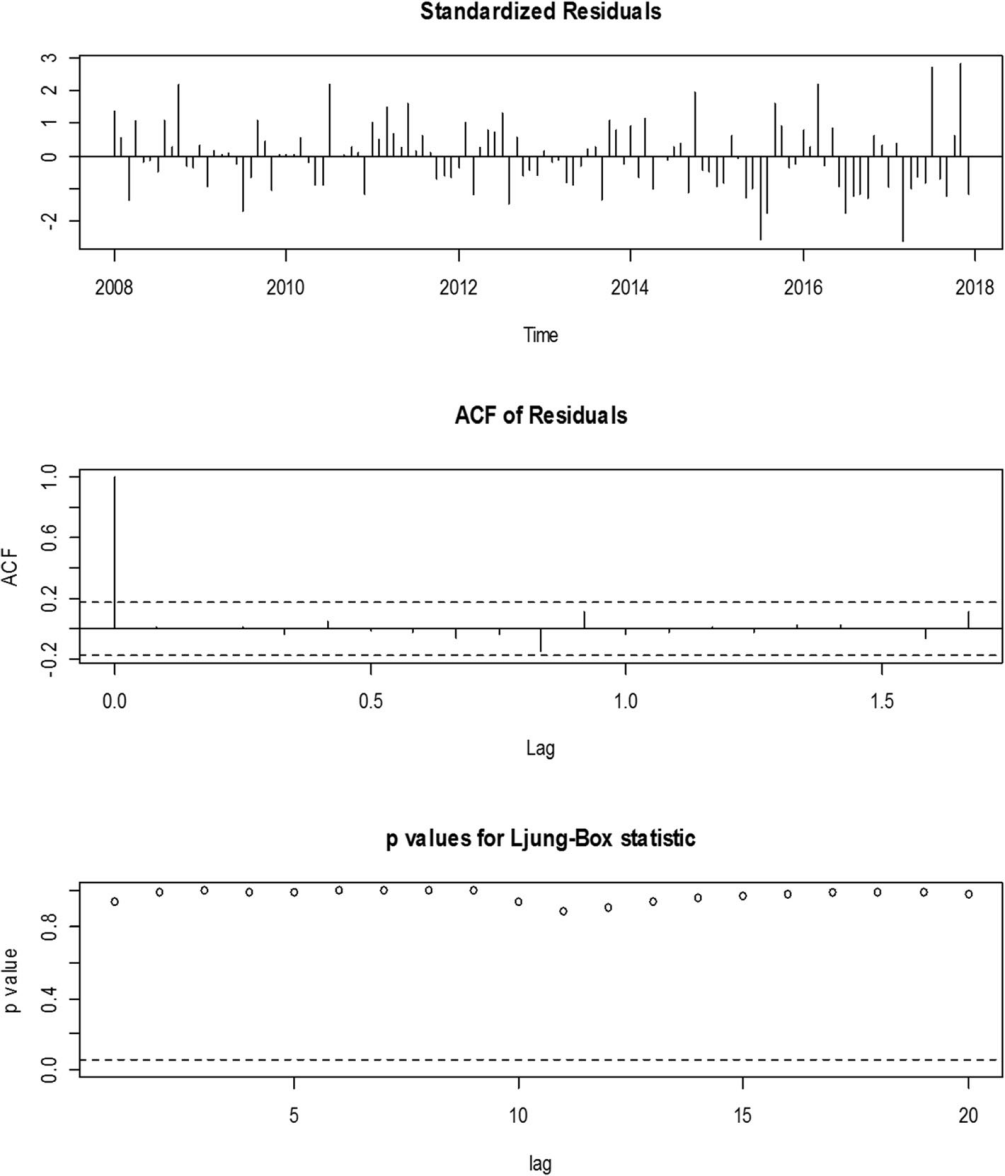
Plots of the standardised residuals (at the top), ACF of residuals (at the middle) and Ljung-Box statistic (at the bottom). The data points in the standardised residuals plot determine the randomness of the residuals for the actual data period (2008–2017) and the forecasted year (2018). The data points in the ACF of the residuals which ranged from -0.2 to 1.0 at various lags assessed the independence of the autocorrelation function (ACF). The data points of Ljung-Box statistic which ranged from 0.0 to 1.0 at various lags represent the *p*-values of the residuals. The horizontal dash lines in the ACF of the residuals and Ljung-Box statistic are the significant bounds

The standardised residuals plot in Fig. 5 (at the top) were random and the lags of the autocorrelation of residuals (middle plot) were all within the significant bounds. The ACF of residuals ranged between − 0.2 and 1.0. All the *p*-values of the Ljung-Box test (Fig. 5 bottom plot) which ranged between 0 and 1 were all above the significant line indicating the residuals were independent (χ^2^ = *8.951; p-value = 0.984*).

### Forecasting

**Fig. 6:**
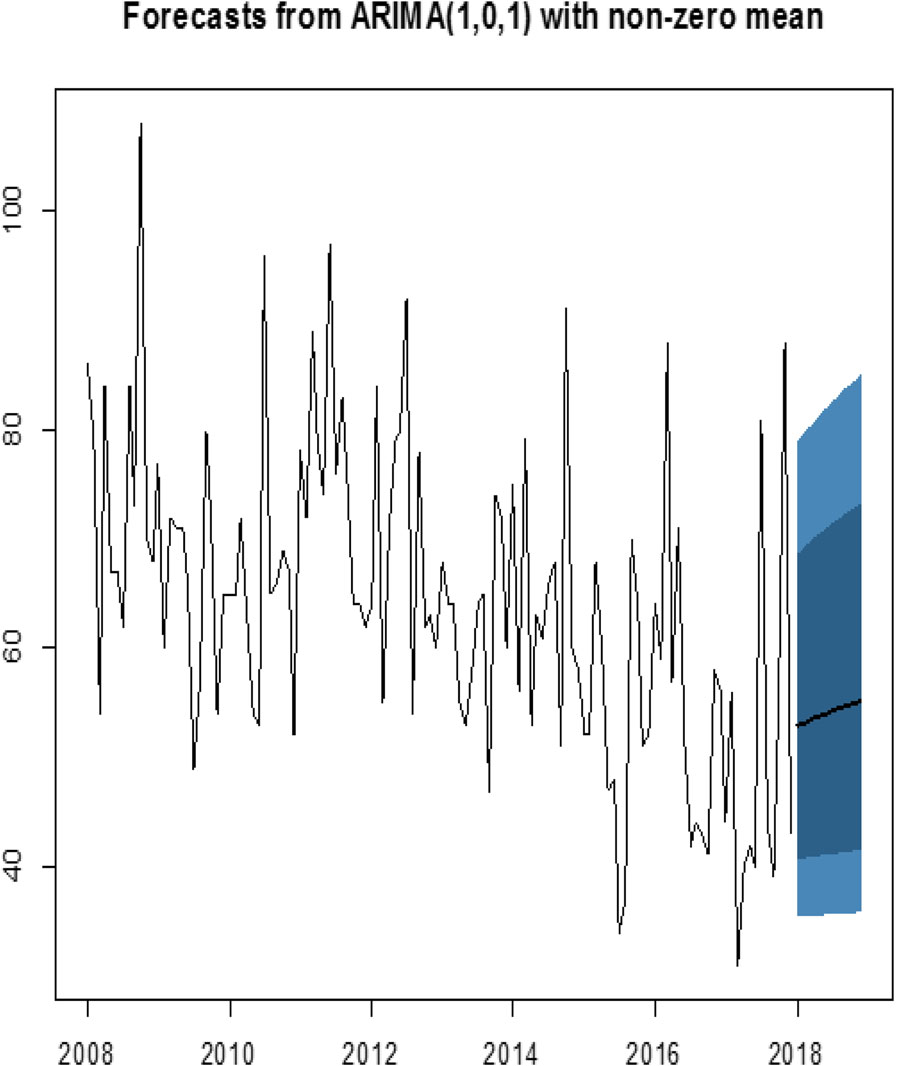
Plot of the actual data and the forecasted values from ARIMA (1,0,1). The data points represent the plot of the data from 2008 to 2017 and the shaded region shows the forecasted figures for 2018

Table 2 showed the monthly (January–December) forecast of tuberculosis cases for the year 2018 which ranged from 53 to 55 with their respective 95% confidence interval. The monthly forecasted TB cases depicted a slow steady rise in the incidence of TB cases for the year 2018 as shown in the line of the shaded region in Fig. 6.

**Table 2:** Forecasted values for the year 2018

Month	Point forecast	95% Confidence interval
January	53	35–79
February	53	36–80
March	53	36–81
April	54	36–81
May	54	36–81
June	54	36–82
July	54	36–83
August	54	36–83
September	55	38–89
October	55	36–84
November	55	36–84
December	55	36–85

### Forecasting accuracy

Table 3 depicts the mean absolute error (MAE) and Mean squared error (MSE) which determined the forecasting accuracy among the competing models. ARIMA (2,0,1) yielded MAE and MSE of 15.08 and 297.25 respectively which produced the minimum errors compared to the other models [i.e ARIMA (1,0,1) and SARIMA (1,0,1)*(1,0,1)_12_].

**Table 3:** Forecasting error

Model	Mean Absolute Error(MAE)	Mean squared Error (MSE)
ARIMA (1,0,1)	15.75	307.92
ARIMA(2,0,1)	15.08	297.25
SARIMA (1,0,1)*(1,0,1)_12_	15.75	300.25

## Discussion

The time series plot of the data showed a general levelled trend although there was a slow downward trend with irregular variations. The series was fairly of the same level between 2008 and 2012 but began to decrease slowly thereafter. Thus, there was no clear evidence of a trend in the series and the mean of the log-transformed of the series was constant and the variance was fairly stable over time. Both the ACF and PACF tailed off to zero indicating stationarity of the series. A test of stationarity using the log-transformed data showed the series was stationary *(ADF = − 3.71, p-value = 0.026***)**, implying that the mean of the TB data is independent of time. This is an evidence of the lack of apparent trend in the series of the log-transformed data.

We found that the ACF showed significant lags at lag 0, lag 1, lag 2, lag 3 etc. and PACF showed significant lags at lag 1 and lag 2. However, lag 1 was selected for both ACF and PACF since they yielded a better estimate of the model than other lags. Hence, ARIMA (1, 0, 1) or ARMA (1,1) model produced the best model for the 10-year TB data.

This model was chosen from among the other models because it has a minimum AIC and BIC compared to the other competing models. The authors found that a plot of the standardized residuals (Fig. 3) was constant over time and the normality test of the residuals(Fig. 4), as well as Ljung-Box test (Fig. 5) depicting the appropriateness of the best-obtained model for forecasting. However, the best model selected does not necessarily give the best results as far as the mean absolute and the mean square errors are concerned (Table 3). The forecasted values in this study exhibit a monthly marginal steady increase in TB incidence for the year 2018.

Various time series models or methods have been used in predicting monthly tuberculosis incidence. ARIMA model has been shown as the best suitable model for predicting TB cases among other forecasting methods such as Moving Average, Artificial Neural Network, Decomposition, linear regression and Holt-winters models [14].

Generally, TB is not known to exhibit seasonality just like malaria, diphtheria, chickenpox, rotavirus, cholera among others [15], yet several studies have investigated the seasonal effect of TB using seasonal ARIMA model showing variations with peaks in the summer, autumn, winter and spring [16–18]. Longitudinal studies by Moosazadeh et al. [1, 19, 20] in Iran on diagnosed tuberculosis cases using Box-Jenkins time series approach yielded seasonal ARIMA (0, 1, 1) (0,1,1) _12_ with peaks in spring and summer. These findings were comparable to a recent national study in China by Wang et al. [21] involving 13 years of monthly TB data which produced the same seasonal model with TB peaks in spring. Another study by Willis et al. in the USA using the Decomposition Time Series method indicated seasonality in TB data with a peak in spring and trough in late fall [22]. Bras et al. [23] used seasonal trend LOESS (STL) to model trend and seasonality of pulmonary tuberculosis (PT) in Portugal. Their findings indicated that SARIMA (2, 1, 0) (0,1,1)_12_ was the best fit for the data and PT incidence peaked in the early spring and trough in winter. A study from South Africa also produced similar tuberculosis seasonality [24].

However, in this study, Box-Jenkins time series approach on monthly TB cases produced non-seasonal ARIMA (1, 0, 1) model even though a peak was observed in the actual data in October and trough in March. These findings support a previous study done in the Ashanti Region of Ghana by Gyasi-Agyei and colleagues [12]. The authors used aggregated TB cases in the region, yet could not determine any seasonal pattern. Therefore, the data in the region was best modelled with ARMA (1, 0) or AR (1).

Most of the studies predicting seasonal variations pertaining to the incidence of TB were done in the developed countries. Explanations regarding seasonal variations are not well recognised but it has been assumed to be attributed to cold weather and living in restricted spaces, which could contribute to the differences between previous studies done and the current study. Most developed countries endure relatively severe cold weather during the year compared to developing countries like Ghana. During such seasons, it has been noted that the incidence of TB is high due to the delicateness of the immune system as a result of low level of Vitamin D production in winter [25]. A decline in sunlight which leads to a drop in Vitamin D may markedly intensify the chances of getting tuberculosis [26]. Also, the chances of TB transmission upsurge in the winter when there is overcrowding, reduced airflow and increased humidity from indoor activities [27].

Another factor that may have accounted for non-seasonality in this study could be a delay in diagnosis or delay in the presentation of the disease. Therefore, the data used in this study may encompass the incorrect time of diagnosis or onset of TB. Most of the previous studies done used aggregated national data, allowing for higher TB cases which may have revealed the seasonal effect in their studies. However, this study was limited to one tertiary referral hospital in Ghana receiving complicated TB cases from primary and secondary healthcare facilities throughout the country. This may, therefore, have had an influence on the non-seasonal behaviour of the data.

Demographic and co-morbid variables such as age, gender, socioeconomic status, HIV/AIDS and diabetes as well as climatic data such as temperature, rainfall and humidity associated with TB transmission were not accounted for in this retrospective study. Hence the forecasted results must be explained with caution but other variables must be included to allow more robust time series models or methods in future studies. The study was conducted in Korle-Bu Teaching Hospital, thus, the results may not be applicable to other settings in Ghana. However, the results of this study may be helpful in putting up a proposition to interpret the changes of the event noticed in order to establish epidemiological surveillance, proper allocation and use of health resources in Ghana.

## Conclusions

There was no trend nor seasonal changes in the Univariate time series data of TB cases at the Korle-Bu Teaching Hospital. Irregular or random fluctuations were observed in the 10-year-data studied. The TB data was best modelled with ARIMA (1, 0, 1) or ARMA (1, 1). The model equation to estimate the expected monthly TB cases at KBTH produced an AR coefficient of 0.971 plus an MA coefficient of − 0.826 with a constant value of 4.127. There was a slow steady increase in the monthly forecasted values for the year 2018. This is essential for developing a hypothesis to explain the dynamics of TB occurrence so as to plan prevention programmes, optimal use of resources and effective service delivery.

## Additional file

Additional file 1: Reported monthly TB cases data set for a ten-year period at the chest clinic-Korle-bu teaching hospital. (XLSX 12 kb)

## Abbreviations

ACF: Autocorrelation Function
ARIMA: Autoregressive Integrated Moving Average
ARMA: Autoregressive moving average
PACF: Partial Autocorrelation Function
PLWH: People Living with HIV
PT: Pulmonary Tuberculosis
TB: Tuberculosis
